# Supplementary material for: Mechanistic interpretation of non-coding variants for discovering transcriptional regulators of drug response
Source: BMC Biol. 2019 Jul 30;17:62. doi: 10.1186/s12915-019-0679-8 (PMC6664756; doi:10.1186/s12915-019-0679-8)

Note S1: Here we describe how we pre-process ChIP-seq data for training purposes.

We used clustered ChIP (version 3) track from ENCODE UCSC as experimental evidence of TF binding. Signal scores, used as measurement of experimental TF binding strength, are collected from ChIP-seq signal files from ENCODE project. We selected 1000 TFBS for each TF as positive sequences using following steps:

1. TF ChIP peaks of GM12878 cell line from TFBS clusters were used.
2. A TF's peak in the TFBS clusters is defined as a High Occupancy Target Region (HOT Region) if there are 6 or more TFs having TFBS overlapping over half of the peak.
3. For each TF, we defined a subset of ChIP peaks from TFBS clusters as function peaks with two criterions. 1) overlap with a protein coding gene's 50kb upstream regulatory region; 2) not a HOT region.
4. We selected the top 1500 peaks with the largest ChIP score for each TF.
5. For each selected peak, signal scores for each base pair in the window were obtained from ChIP-seq signal file. The maximum signal score was used as a measurement of TF binding strength at this window. The window was then modified to be a 501bp window around the position with the maximum score (250bp upstream and 250 bp downstream). Top 1000 peaks with the largest signal score were selected to be positive windows where the corresponding TF binds.
6. Negative windows were defined as open chromosome region that are able to be bound by other TFs and show no evidence of binding of the target TF. Negative windows for TF_i were selected as follows: 1) Take a union of all positive windows of all TF_j $(j \neq i)$. 2) Remove windows with signal scores greater than the mean of the 1000 positive window signal scores of TF_i. 3) Randomly select 1000 windows from this union as negative windows of TF_i.

ENCODE clustered ChIP track contains ChIP-seq data for 62 TFs for GM12878. After the pre-processing steps mentioned above, the number of TFs having enough data decreased to 37. A dataset with sequences and signal scores of these 2000 windows were obtained for each TF and used for training and testing.

Note S2: eQTL evaluation

Besides ASB evaluation, we also evaluated TFBS-SNP impact prediction of four methods by asking if the 'binding-change SNPs' are enriched for eQTLs using hypergeometric tests. We evaluated 'binding-change SNPs' predicted by four methods: Delta-MOP, Delta-gkmSVM, Delta-STAP and Delta-LLR and compared them with a set of same number of randomly selected SNPs that covered by the TF's ChIP peak. The scatter plots show pairwise comparison of each method (Delta-MOP, Delta-gkm-SVM, Delta-STAP and Delta-LLR) and its corresponding random test. For all four methods, the overall enrichments were similar no matter the TF binding predictors were used or not.

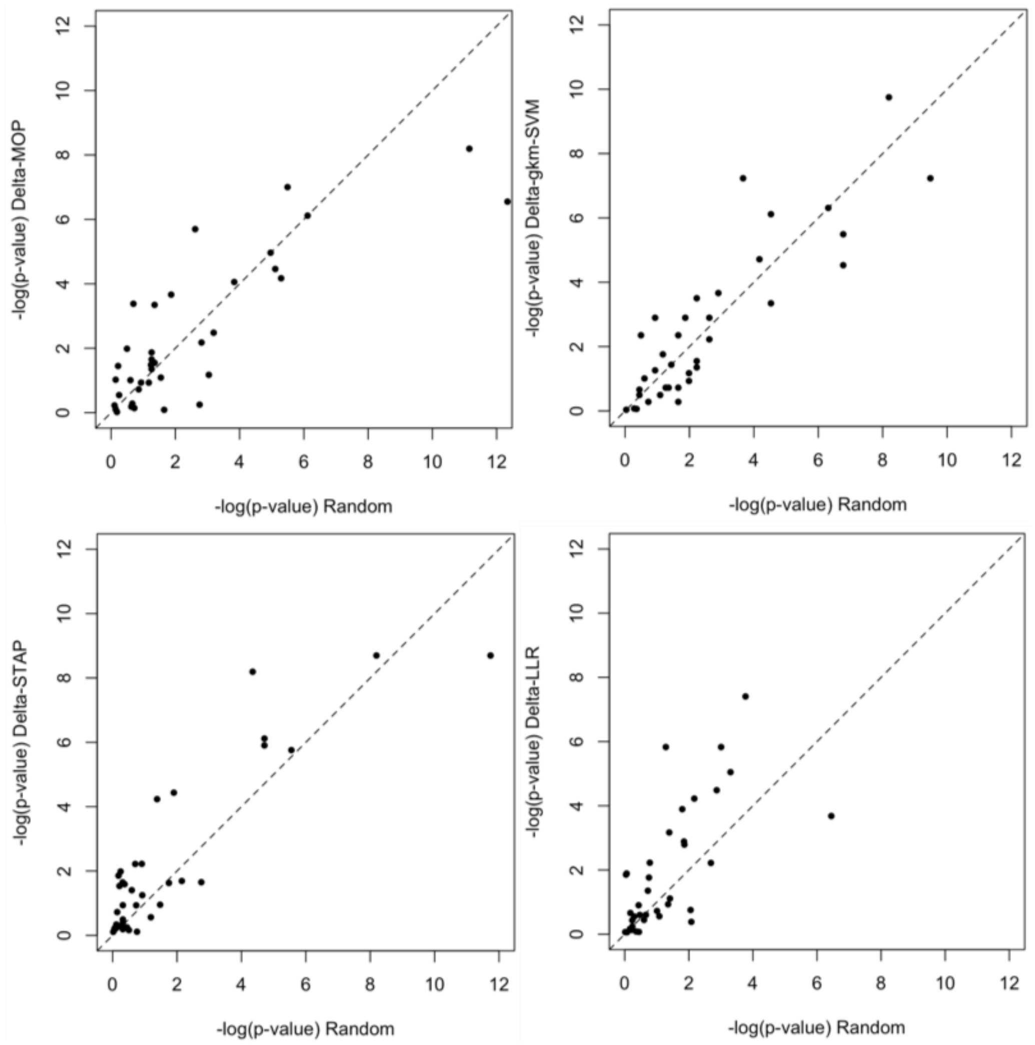

Note S3: Here we list and discuss the literature evidence found for the 38 significant TF-Drug pairs identified in the TF-Drug analysis. For each TF-Drug pair, we provide an evidence code and a brief summary of the evidence. We considered five types of evidence code.

1. **Direct**: A direct evidence is usually exhibited by TF knockdown or impact on drug response through TF binding or expression.
2. **Regulation of Drug Action**: Target TF has regulatory impact on drug action.
3. **Regulatory Target Direct**: Target TF is a regulator of a gene with direct evidence.
4. **Sibling Drug Evidence**: Target TF has direct evidence for a sibling drug.
5. **Complex Partner Direct**: Target TF is a complex partner of a protein with direct evidence.
6. **Sibling of Direct**: Target TF is a sibling of a protein with direct evidence.
7. **Indirect and No Evidence**: Other types of evidence than the first five are classified as indirect evidence. Indirect evidences are not considered as support for the TF-Drug pair.

1 ELF1 – Rapamycin

Evidence Code: Indirect

Evidence: The TF ELF4 is a part of the mTOR pathway and its expression is regulated by mTOR in CDH8+ cells; rapamycin inhibits mTOR. ELF4 is a paralog of ELF1 [PMID 20802152].

2 ELF1 – Epirubicin

Evidence Code: Direct

Evidence: TF Knockdown [PMID 26503816].

3 MAX – CDDP

Evidence Code: Complex Partner Direct

Evidence: MXD1, a member of the Myc-Max-Mxd family, has been shown to induce CDDP (cisplatin) resistance in hypoxic U 2OS and MG 63 cells [PMID 28543796]. Mxd1 (MAD) is a well-known dimerization partner of MAX [PMID 9528857, PMID 11602341], and the reported association of MAX with CDDP is thus likely to reflect the previously demonstrated Mxd1-CDDP relationship.

4 SIN3A – CDDP

Evidence Code: Complex Partner Direct

Evidence: SIN3A is part of the SIN3A-HDAC complex that functions as a repressor and is associated with diseases including cancer [PMID 29741645], and HDAC inhibitors are known to potentiate CDDP activity [PMID 21278889, PMID 26024393, PMID 24980825].

5 SP1 – Carboplatin

Evidence Code: Direct

Evidence: PMID 24122978.

6 ELF1 – Doxorubicin

Evidence Code: Direct

Evidence: TF knockdown [PMID 26503816].

7 NFYB – 6TG

Evidence: [no TF in database for this drug] NFYB, when induced by E2F1, attenuates apoptosis. Nothing specific to 6TG.

8 MAX – 6TG

Evidence: [no TF in database for this drug] MAX is a heterodimeric partner of c-Myc, which is known to affect mutation rate in the HGPRT gene; the latter in turn is a critical component of 6-TG sensitivity [PMID 12584560].

9 SIN3A – MPA

Evidence: [no TF in database for this drug]

10 ELF1 – Carboplatin

Evidence Code: Regulatory Target Direct; Sibling Drug Evidence

Evidence: ELF1 is one of the top 20 molecular features associated with response to the decitabine: carboplatin combination, as per CTRP database (PharmacoDB). Also, ELF1 is an ETS family transcription factor and other TFs of this family, such as ETS-1, have been shown to be involved in resistance to platinum drugs [PMID 24624361]. ELF1 is also believed to regulate BRCA2, which in turn is a key determinant of response to platinum therapy [PMC3939430].

11 NFYB – 6MP

Evidence Code: No evidence

12 MXI1 – CDDP

Evidence Code: Sibling of Direct

Evidence: MXI1, also known as MXD2, is a member of the MXD1 family, and a closely related member of this family – MXD1 – has been shown, via knock-down assays, to affect CDDP resistance in hypoxic conditions via repression of PTEN [PMID 28543796].

13 YY1 – CDDP

Evidence Code: Direct

Evidence: YY1 knock-down in non-small cell lung cancer (NSCLC) cells increased sensitivity to CDDP [DOI:10.1096/fasebj.30.1_supplement.699.8] though no effect was seen in ovarian cancer cell lines [PMID 19208743].

14 SP1 – CDDP

Evidence Code: Direct

Evidence: SP1 inhibition by tolfenamic acid (TA) was found to increase CDDP response in epithelial ovarian cancer (EOC) cell lines [PMID 27581819].

15 ELF1 – CDDP

Evidence Code: Direct

Evidence: As noted above, ELF1 is believed to regulate BRCA2, which in turn is a key determinant of response to platinum therapy [PMID 24624361]. Also, ELF1 binding to DNA was found to increase DNA damage upon cisplatin treatment [PMID 28607063], suggesting that the TF may modulate cisplatin activity.

16 SIN3A – 6TG

Evidence Code: No Evidence

17 PML – Epirubicin

Evidence Code: Sibling Drug Evidence

Evidence: [PMID 22110895] See PML-Doxorubicin association below.

18 REST – Rapamycin

Evidence Code: Regulatory Target Direct

Evidence: A previous study showed that REST exerts regulatory control over the ‘mammalian Target Of Rapamycin’ (mTOR) signaling pathway [PMID 25524378], which is the pathway

mediating Rapamycin induced apoptosis. mTOR-Rapamycin is a ‘direct’ evidence pair [PMID 19568796].

19 REST – 6MP
Evidence Code: No Evidence

20 NFYB – Epirubicin
Evidence Code: No Evidence

21 SP1 – Epirubicin
Evidence Code: Sibling Drug Evidence
Evidence: SP1 has been found in various studies to be associated with the activity of Doxorubicin (see below), a drug closely related to Epirubicin (of the anthracycline family). The association between SP1 and Doxorubicin was also reported as significant by our analysis, see below.

22 BHLHE40 – TMZ
Evidence Code: Indirect
Evidence: The apoptosis inhibitor BHLHE40, also known as DEC1, has been shown to be predictive of TMZ response in glioma patients [PMID 27840944].

23 PBX3 – Epirubicin
Evidence Code: No Evidence

24 PML – Doxorubicin
Evidence Code: Direct
Evidence: The promyelocytic leukemia (PML) protein was shown to regulate DNA modification in response to Doxorubicin and knockout of PML was found to abolish Doxorubicin-induced DNA modification and to regulated inhibition of cell proliferation by the drug [PMID 29735542].

25 MXI1 – 6MP
Evidence Code: No Evidence

26 SP1 – Oxaliplatin

Evidence Code: Regulation of Drug Action

Evidence: A study of oxaliplatin response in esophageal cancer cell lines showed that survivin may be a key target of the drug, and that downregulation of survivin by the drug is mediated by SP1, thus arguing in favor of a major role for SP1 in oxaliplatin response [PMID 17949450].

27 MAX – 6MP

Evidence Code: Indirect

Evidence: Evidence Code MAX is a heterodimeric partner of c-Myc, which is known to affect mutation rate in the HGPRT gene [PMID 12584560]; the latter in turn is a critical component of 6-MP resistance (https://www.sciencedirect.com/science/book/9780123725516).

28 REST – Carboplatin

Evidence Code: No Evidence

29 NFYB – Doxorubicin

Evidence Code: No Evidence

30 MAX – Epirubicin

Evidence Code: Complex Partner Direct

Evidence: We note that various homo- and hetero-dimers of the MAX family proteins including MAX-MAX and MYC-MAX have similar binding preferences and compete for each other's sites, so our report of an association with MAX may equally well represent an association with MYC-MAX. Another study showed that cMyc, an important binding partner of MAX, attenuates the effect of Doxorubicin [DOI: 10.3724/SP.J.1206.2010.00048], a drug closely related to Epirubicin. Also, Doxorubicin was shown to disrupt MYC-MAX interaction [PMID 22132187] and thus affect DNA binding by MYC-MAX, suggesting that regulatory effects of MYC-MAX may underlie Doxorubicin response variation.

31 ATF2 – Fludarabine

Evidence Code: Indirect

Evidence: The drug Fludarabine, a purine anti-metabolite, shares properties similar to cladribine and gemcitabine. A gemcitabine combination therapy downregulated ATF2 phosphorylation and activated SAPK/JUNK activation, which in certain cell types yields ATF2-cJun heterodimerization [PMID 24413065]. In another study, fludarabine treatment, in a

cocktail of therapies, led to the Inhibition of the AKT pro-survival pathway and activation of the JNK-ATF2 pathway; fludarabine therapies seemingly increased the concentration of P-ATF2 [PMID 25704054].

32 BHLHE40 – Epirubicin
Evidence Code: Direct
Evidence: BHLHE40, also known as DEC1, is has been shown to induce cell cycle arrest and premature senescence on Doxorubicin treatment in MCF7 cells [PMID 18025081]. The mechanism for this was further explored in [PMID 18556654].

33 SP1 – Doxorubicin
Evidence Code: Regulation of Drug Action
Evidence: SP1 activity is increased in Doxorubicin-resistant HL 60 leukemia cells [PMID 2204818], and SP1 expression levels are predictive of long term prognosis in triple negative breast cancer patients under Doxorubicin treatment [PMID 27545642]. Moreover, a recent study showed that insulin pretreatment protects against Doxorubicin induced apoptosis by SP1-mediated transactivation of survivin [PMID 26271039].

34 MXI1 – Carboplatin
Evidence Code: Indirect
Evidence: MXI1, also known as MXD2, is a member of the MXD1 family, and a closely related member of this family – MXD1 – has been shown, via knock-down assays, to affect cisplatin resistance in hypoxic conditions via repression of PTEN. Cisplatin is a platinum drug closely related to carboplatin, believed to have similar resistance mechanisms [https://www.croh-online.com/article/S1040-8428(07)00034-0/pdf].

35 ATF2 – Everolimus
Evidence Code: No Evidence

36 PAX5 – Hypoxia
Evidence Code: Indirect
Evidence: Hypoxic conditions increase Pax5 expression in KMS-12BM cells [PMID 24126540].

37 SIN3A – MTX

Evidence Code: Indirect

Evidence: The SIN3A repressor complex is a master regulator of the transcriptional activity of STAT3 [PMID 22783022], and STAT3 has been shown to be directly involved in MTX sensitivity [PMC4801443].

38 PBX3 – Doxorubicin

Evidence Code: No Evidence

Note S4: Here we list and discuss the literature evidence we found to show the impact of mediator genes on Drug-induced apoptosis pathway. We looked at the mediator genes identified in the ELF1-Doxorubicin enrichment test.

Gene	Evidence
ANKRD52	ANKRD52, interacting with PP6, lead to the dephosphorylation of PAK1 [DOI: 10.1158/1538-7445.AM2018-5508], which can protect cell from apoptosis through bcl-2 involved pathway [DOI: 10.1517/14728222.2010.492779].
ATM	ATM is part of the ATM/TP53 pathway and plays an important role in the activation of TP53 [PMID 21494257].
ATP6V0D1	ATP6V0D1 is a subunit of V-ATPase (NCBI Gene: ATP6V0D1). Inhibition of V-ATPase activity can lead to cell death [PMID 22360554, PMID 27335445, PMID 23168408].
B4GALT2	In HeLa cells, B4GALT2 is identified as a target gene of p53-mediated apoptosis [PMID 18211920] and its overexpression can induce cell apoptosis [PMID 23744354].
BAK1	
COMMD9	Decreasing the expression of COMMD9 leads to the decreasing of E2F1 activation which enhances p53-mediated apoptosis [DOI: 10.1016/j.cellsig.2016.11.016].
EIF4A3	EIF4A3 plays an important role in the alternative splicing of BCL2L1/BCL-X and probably also other apoptotic genes [PMID 22203037]. Knocking down of EIF4A3 increases the expression of BCL-Xs in HeLa cells [DOI: 10.1128/MCB.06130-11].
IPO13	IPO13 mediates the nuclear translocation of PDCD5 [PMID 26077467], which promotes apoptosis through HDAC3/p53 pathway [PMID 26775586].
miR6734	miR6734 up-regulates the expression of p21 and induces cell arrest and apoptosis [PMID 27509128].
NABP2	NABP2 plays an important role in the DNA damage response [PMID 27273218]. It can also regulate the stability and transcriptional activity of p53 [PMID 23184057].
NEURL4	NEURL4 regulates the activity of p53 [PMID 28977907].
REXO4	REXO4 is also known as hPMC2, which regulates the expression of p21 [PMID 27589731].
TFIP11	TFIP11 is also known as TFIP-1 (NCBI Gene: TFIP11). Knocking down TFIP induces apoptosis through caspases 3 and caspase 9 [PMID 26354852].
TMEM86B	TMEM86B has protein-protein interaction with BCL2L13 (STRING) [http://version10.string-db.org/].
URI1	URI1 deficiency is associated with p53-mediated apoptosis [PMID 27105489].

Note S5: Accuracy of TF binding strength prediction using two motifs

We simulated the case where only two motifs for a TF are available.In particular:

1) we selected six TFs arbitrarily, each of them having 5 or more motifs;
2) for each TF, we selected two motifs at random and pretended that these were the only available motifs;
3) we then evaluated MOP and STAP, exactly as in the paper, on this simulated scenario of a TF with two motifs, and compared their performance (correlation coefficient);
4) we repeated steps 2-3 ten times for each TF and summarized the results in scatter plots similar to Figure 2b, except that each scatter plot now corresponds to a single TF and shows 10 points representing random subsets of two motifs of the TF.

We noted that for three of the six TFs (viz., ATF2, EGR1, and ZNF143), MOP showed an improvement over STAP even with two available motifs, often with several such choices of two motifs. For the TF SPI1, MOP with two randomly selected motifs gave results comparable to STAP, even though MOP with all available 7 motifs showed improved performance over STAP. Finally, for two of the TFs – BATF and ELF1 – MOP with two motifs was of comparable accuracy to STAP, as was MOP with all available motifs.

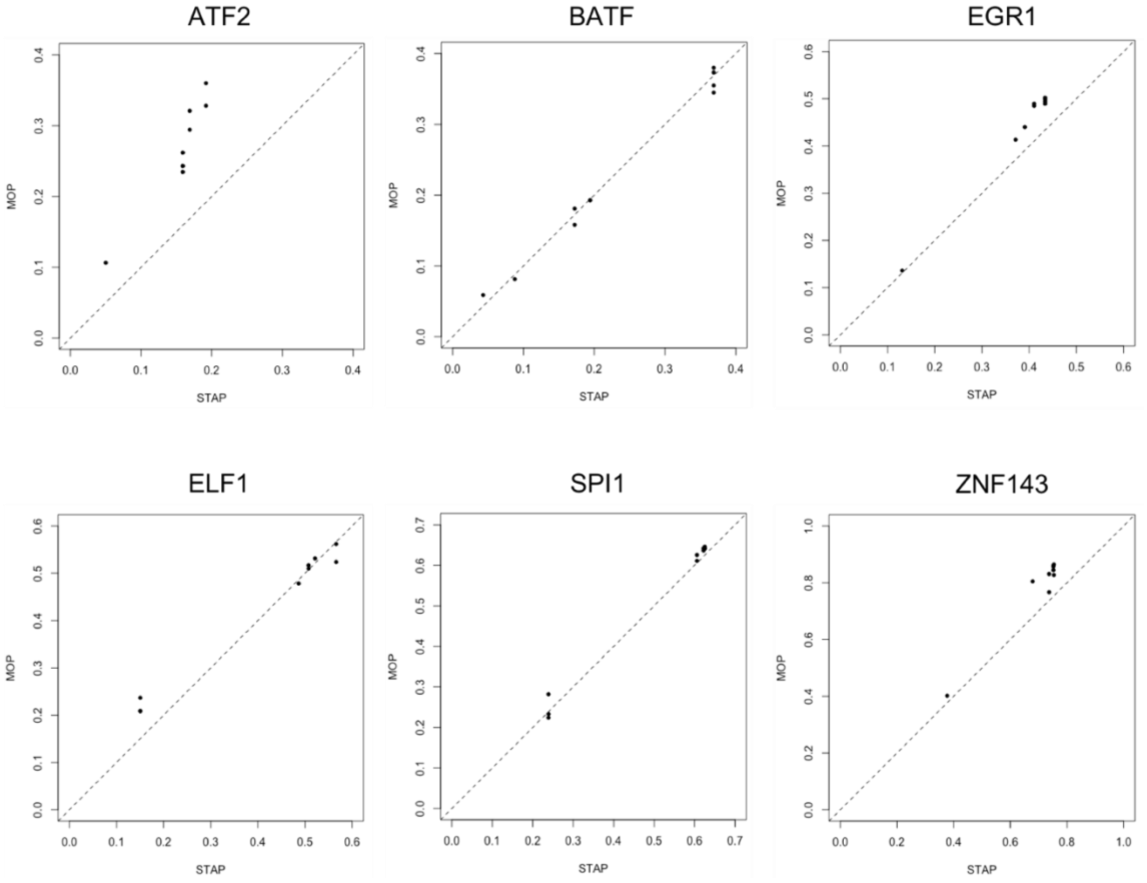

Note S6: TFBS-SNP impact prediction comparison between Delta-MOP and Delta-ls-gkm.

We compared the Delta-MOP method with Delta-ls-gkm in the ASB evaluation using the same procedure as used in the comparison among Delta-MOP, Delta-STAP and Delta-gkm-SVM, which is described in the Result section. Each point in scatter plots below corresponds to an ASB data set corresponding to a single TF. For each TF, ls-gkm was trained on the same training set of ChIP peaks of that TF that MOP was trained on. Delta-ls-gkm and Delta-MOP have similar average AUROC (left panel, p-value of paired t-test > 0.53) and similar average AUPRC (right panel, p-value of paired t-test > 0.19) on the evaluated data sets.

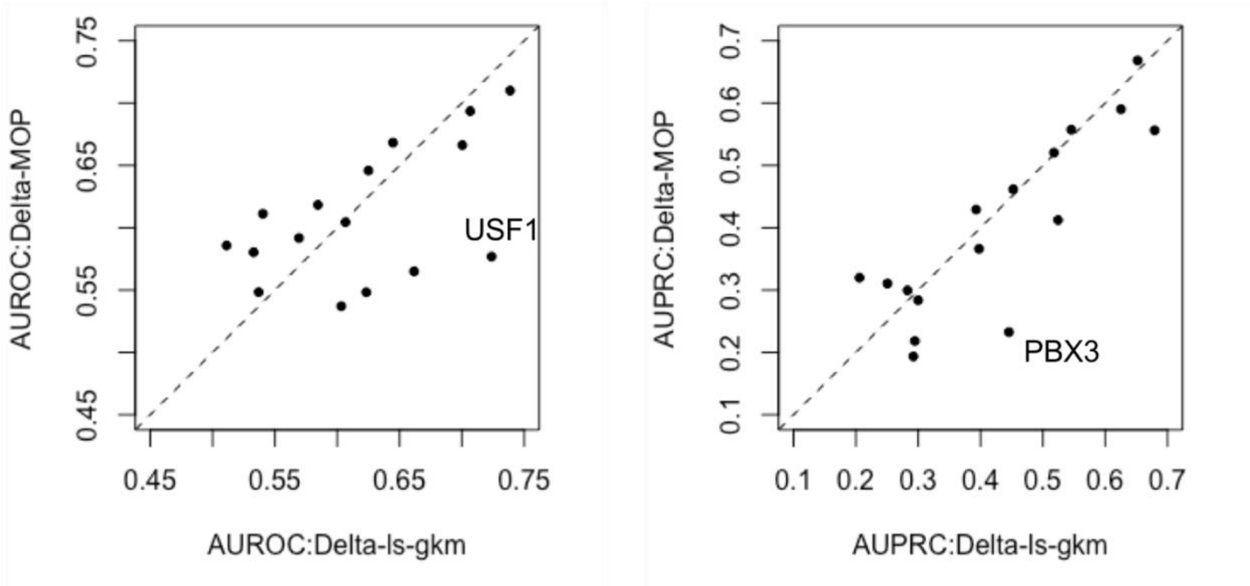

Note S7: TFBS-SNP impact prediction comparison between Delta-MOP and DeepSEA.

We compared the Delta-MOP method with DeepSEA, as a representative of the deep learning approach, in the ASB evaluation using the same procedure as used in the comparison among Delta-MOP, Delta-STAP and Delta-gkm-SVM, which is described in the Result section. Each point in scatter plots below corresponds to an ASB data set corresponding to a single TF. Binding impact scores of DeepSEA were downloaded from Wagih et al. (doi: https://doi.org/10.1101/253427), and were not based on training of the model on the same training set as that used for training MOP. There is no clear 'winner' between the two, with their average AUROC being similar (left panel, paired t-test p-value > 0.13) and their average AUPRC being similar (right panel, paired t-test p-value > 0.09) on the evaluated data sets.

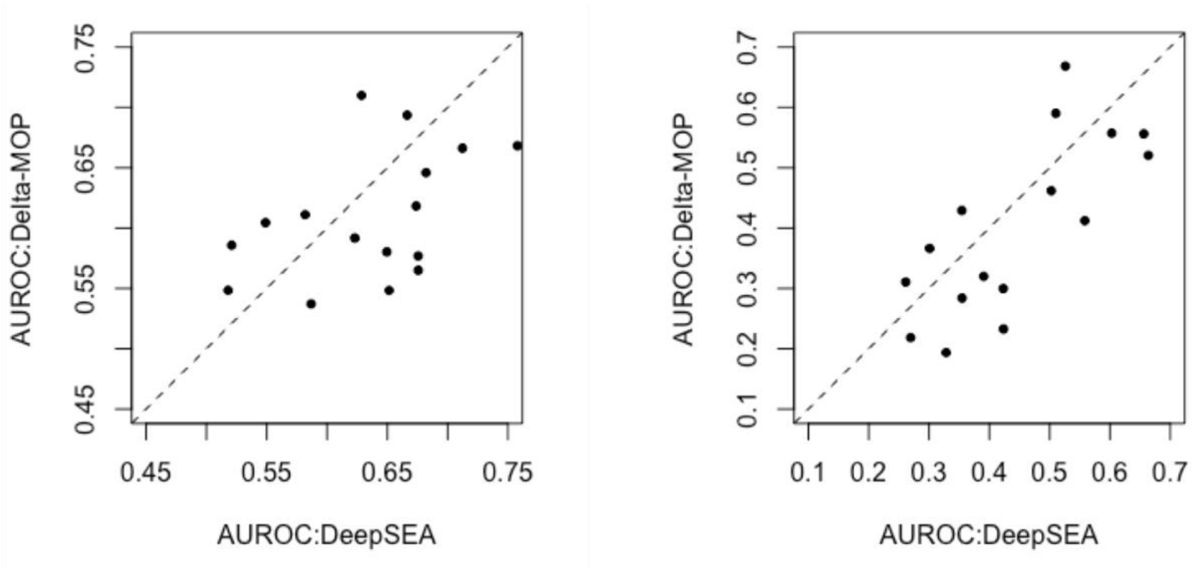

Supplement: Supplementary file 1 — Supplementary notes. This file contains seven supplementary notes, with each accompanied by short descriptions. (PDF 637 kb) [file 12915_2019_679_MOESM1_ESM.pdf]
